# Influence of cold atmospheric-pressure-plasma in combination with different pretreatment methods on the pull-off tensile load in two-piece abutment-crowns: an in-vitro study

**DOI:** 10.1186/s12903-023-02880-9

**Published:** 2023-03-30

**Authors:** Carolin-Isabel Görgen, Kawe Sagheb, Karl Martin Lehmann, Irene Schmidtmann, Stefan Wentaschek

**Affiliations:** 1 Department for Prosthetic Dentistry and Materials, University Medical Centre, Augustusplatz 2, 55131 Mainz, Germany; 2 Institute for Medical Biostatistics, Epidemiology and Informatics, University Medical Centre, Obere Zahlbacher Str. 69, 55131 Mainz, Germany

**Keywords:** Plasma treatment, Zirconia, Titanium, Resin cement, Pull-off tensile load, Abutment crown

## Abstract

**Background:**

In implant prosthetic dentistry, the adhesive connection of individualized ceramic crowns and prefabricated titanium bases leads to several benefits. However, the durability of the bonding could be a weak point and especially depends on sufficient surface pretreatment. Cold atmospheric-pressure plasma (CAP) is a pretreatment method that should improve the surface properties without physical damage. Thus, the purpose of this study was to investigate the influence of CAP treatment on pull-off tensile load in two-piece abutment crowns.

**Methods:**

Eighty zirconia crowns and titanium bases were divided into eight groups (n = 10) according to their surface pretreatment prior to cementation with Panavia V5: no treatment (A); sandblasting (B); 10-MDP primer (C); sandblasting and primer (D); CAP (AP); sandblasting and CAP (BP); CAP and primer (CP); sandblasting, CAP and primer (DP). The specimens were thermocycled (5°/55°, 5000 cycles), and then the pull-off tensile load (TL) was measured. Statistical analyses were performed using three-way ANOVA with Tukey post-hoc and Fisher’s exact tests.

**Results:**

The results showed that the TL was highest in group D (p < 0.0001). Some combinations of different treatments led to effects that were greater than the sum of the individual effects. These effects were modified by interactions. Only in combination with primer, CAP treatment had a small but positive significant effect (group CP vs. C and CP vs. AP, p < 0.0001) which however did not come close to the strong interaction effect that resulted from the combination of sandblasting and primer.

**Conclusion:**

Within the limitations of this study, CAP treatment cannot be recommended in this specific field of indication due to its unreliable influence on TL in combination with other pretreatment methods.

## Background

In order to unite aesthetic, biological and mechanical advantages in implant prosthetic dentistry, materials such as titanium and ceramics are often combined. Due to its outstanding wear resistance and flexural strength after the final sintering process, zirconia is particularly suitable for prosthetic restorations. In a systematic review, Pjetursson et al. showed a 5-year survival rate of 97.6% and a complication rate of 16.2% for implant-supported single-tooth zirconia restorations [1]. Moreover, the development of computer-aided-design/ computer-aided-manufacturing (CAD/CAM) systems offers an easy and convenient method to design and process mesostructures or crowns of zirconia based on individual aesthetic demands, notably when adhesively connected to prefabricated titanium inserts. Due to their assembled internal connection to the respective implant system, the milling process of this complex geometry can be omitted. Furthermore, fractures and abrasion due to the mixture of materials inside the titanium implant can be reduced, as to be expected with monolithic one-piece zirconia abutments [2–4]. However, only the titanium insert is connected to the implant with a screw which is why the adhesive connection is of particular importance for the restoration’s longevity. The stability of the bonding is affected by several factors which must be optimally coordinated and adapted to one another. Among others, the choice of adhesive cement type, the height of the titanium base, the luting gap size and the surface treatment of the two abutment components are discussed as limiting factors for retention force in two-piece hybrid abutment crowns [5]. In particular, the search for the optimal surface treatment of titanium and zirconia is of major interest, due to some material limitations. In dental technology, there are many options for surface pretreatment, such as chemical, mechanical or mechanochemical processes to roughen and activate the surfaces [6–8]. Invasive physical methods such as laser irradiation have also been considered [9, 10]. The previous gold standard for both titanium and zirconia was sandblasting and the application of an adhesive phosphate primer [11, 12]. However, there is always a risk that the surfaces will be damaged through incorrect handling which can ultimately lead to the premature failure of the restoration. Furthermore, in order to avoid the use of potentially toxic chemicals, research on a gentle but effective method for surface treatment is required.

Plasma technology is a conventional method in the automotive industry and aviation engineering to prepare various materials for adhesive bonding by improving the surface characteristics, such as surface energy and wettability, without damage or changes to the intrinsic properties of the material [13, 14]. Among all plasma types, cold atmospheric-pressure plasma (CAP) generated by ambient air has several advantages for use in the dental laboratory. In particular, the plasma device “piezobrush®PZ2”, created by relyon plasma GmbH, does not require any vacuum equipment or inert gas supply to generate CAP and is suitable for processing small components. Several studies have already examined the impact of CAP on the shear and tensile bond strength between zirconia and resin cements [15–17]. Thus far, the effect of CAP on pull-off tensile load in two-piece hybrid abutment crowns has only rarely been examined, and only when applied to the zirconia surface [18]. Meanwhile, no other study has applied CAP to both abutment components and examined the influence on both materials simultaneously. Accordingly, the purpose of this study was to investigate the impact of CAP treatment of both titanium and zirconia in combination with other pretreatment methods on the pull-off tensile load in two-piece hybrid abutment crowns. The null hypothesis was that additional CAP treatment does not affect the pull-off tensile load between the zirconia crown and the titanium base.

## Methods

### Preparation of specimens

**Fig. 1:**
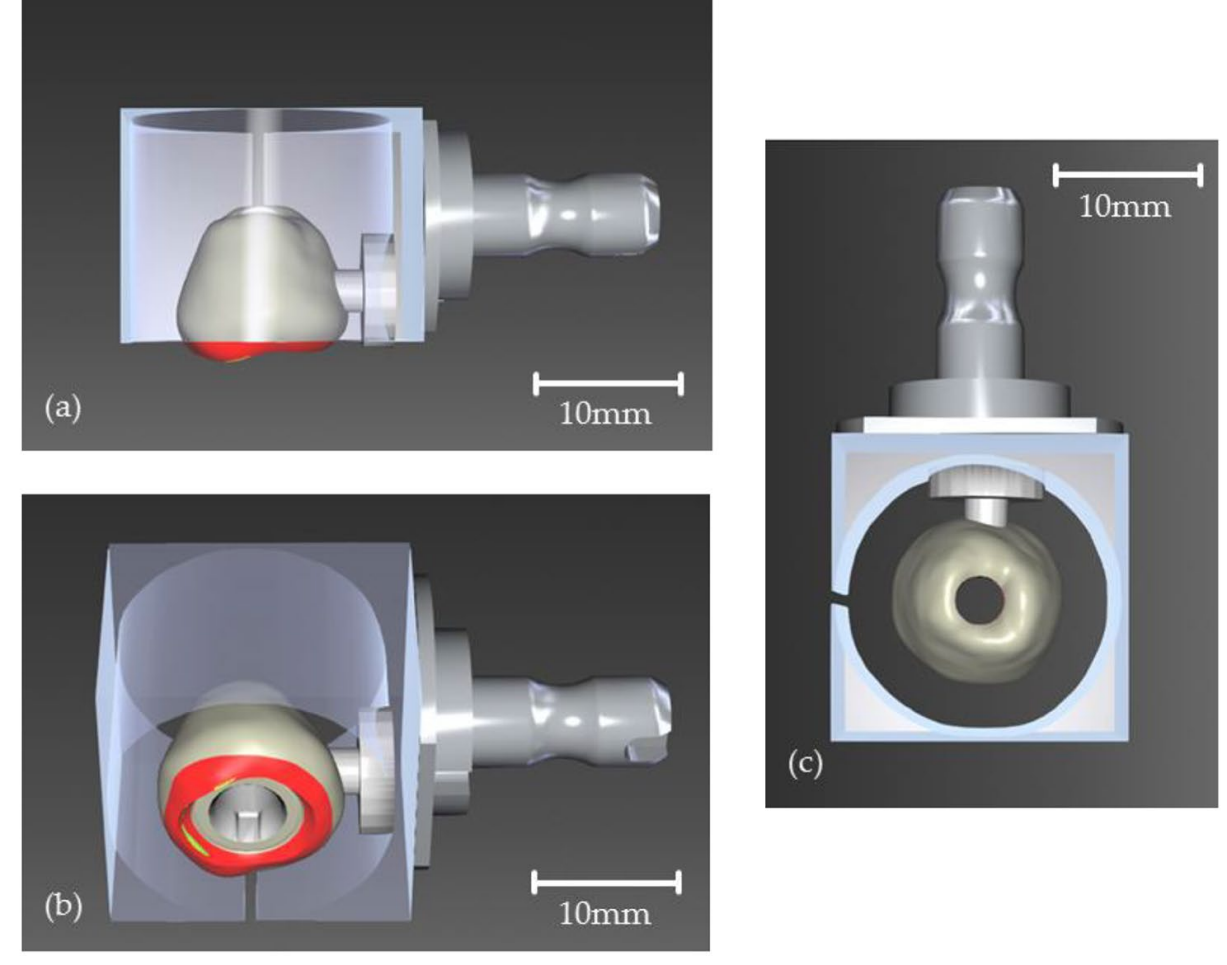
CAD and position of the crown in the virtual zirconia block before CAM. The red and yellow areas of the crown design are intentionally positioned outside the block and are therefore not milled in order to flatten the lower surface of the crown. (a) View from the side. Without the red area, the crown will be flat. (b) View from below with the pre-milled inner connection to the titanium base with anti-rotation lock on the inside of the crown. (c) View from above. The hole in the center represents the pre-milled screw channel of the crown

Eighty zirconia crowns were designed using the CEREC software (CEREC SW 5.0, Sirona Dental Systems GmbH, Bensheim, Germany). The design was intentionally chosen not to be anatomical in order to form a flat lower surface for the pull-off test. Figure 1a-c show the design and position of the crowns within the virtual block. The red areas could not be implemented by the milling machine, so the crown received a flat surface which corresponded to the outer side of the block. The crowns were fabricated from pre-sintered zirconia blocks (inCoris ZI meso F2 L, Sirona Dental Systems GmbH, Bensheim, Germany) and then sintered according to the manufacturer’s instructions. The internal connection of the crown to the titanium base (Ti-Base NB RS 4.3 L, Sirona Dental Systems GmbH, Bensheim, Germany) did not need to be milled, as the connection geometry was prefabricated in both components. Therefore, the cement gap size could be assumed to be the same in all crowns.

**Fig. 2:**
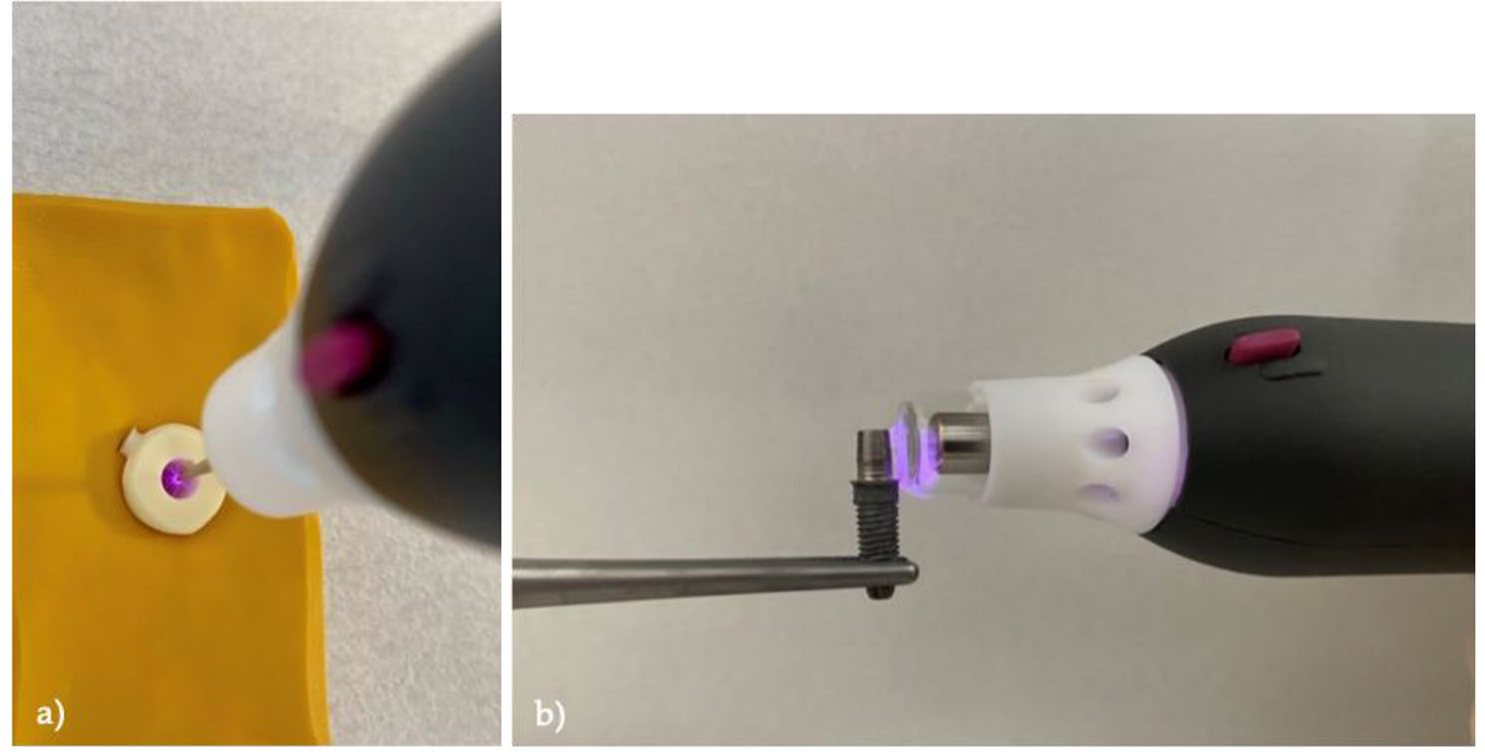
Plasma treatment procedure with “piezobrush PZ2” plasma device to (a) the inner surface of the crown (the crown is fixed in a yellow silicone mold), (b) the titanium base surface (the Ti-base is screwed onto an implant which is fixed in a metal clamp)

Eight different surface treatment protocols were performed on the inner surfaces of ten crowns per group, as well as on the outer surfaces of the associated ten titanium bases (Table 1). Group A had no surface treatment except for alcohol disinfection. Sandblasting (SB) was carried out vertically to the surfaces using a dental sandblaster (P-G 400, Harnisch + Rieth GmbH & Co. KG, Winterbach, Germany) with 50 μm aluminum oxide particles (Al_2_O_3_) (Plurakorund, Pluradent AG & Co. KG, Offenbach, Germany) under a pressure of 1.0 bar and at a distance of 10 mm for approximately 10 s (Groups B, BP, D, DP). The primer (Clearfil Ceramic Primer Plus, Kuraray Medical Inc., Okayama, Japan), containing 10-Methacryloyloxydecyldihydrogen-phosphate (10-MDP), was evenly applied to the surfaces with a small brush and they were carefully blown dry after 10 s (Groups C, CP, D, DP). In groups AP, BP, CP and DP, cold atmospheric-pressure plasma (CAP) was used in addition to previous surface treatments. The plasma was generated using a plasma device (piezobrush PZ2, relyon Plasma GmbH, Regensburg, Germany) operated with ambient air (gas flow rate: 20 L/min; max. plasma and substrate temperature: 50 °C; input voltage: 12–24 V; max. output voltage: 15 kV; max. operating power: 8.0 W). The inner surface of the crowns was treated statically with a special needle nozzle for non-conductive materials in a parallel alignment at a distance of 2 mm for 15 s (Fig. 2a). The titanium bases were treated with a nearfield nozzle for conductive materials. Due to the curved surface of the titanium base, a dynamic treatment was necessary. Therefore, the nozzle was aligned perpendicular to the surface and manually rotated around the titanium base with a distance of 0.5 to 2.0 mm at an even speed of 10 to 20 mm/s for at least 30 s (Fig. 2b).

**Table 1:** Study design and test groups

	Ti-Base (n = 80)			Crown (n = 80)		
Group	SB	CAP	Primer	SB	CAP	Primer
**A (n = 10)**	no	no	no	no	no	no
**AP (n = 10)**	no	yes	no	no	yes	no
**B (n = 10)**	yes	no	no	yes	no	no
**BP (n = 10)**	yes	yes	no	yes	yes	no
**C (n = 10)**	no	no	yes	no	no	yes
**CP (n = 10)**	no	yes	yes	no	yes	yes
**D (n = 10)**	yes	no	yes	yes	no	yes
**DP (n = 10)**	yes	yes	yes	yes	yes	yes

### Bonding of Ti-base and crown

In order to prevent the resin cement from flowing into the Ti-base and therefore obstructing the way to the implant screw, the screw channel was closed with a foam pellet. After that, self-adhesive Bis-GMA/TEGDMA-based resin cement (Panavia V5, Kuraray Medical Inc., Okayama, Japan) was applied to both surfaces and then the complex was placed in an auxiliary tool (CLIP by Hans-Jürgen Joit, HPdent GmbH, Gottmadingen, Germany). This tool could guarantee standardized pressure in all specimens and simplify the handling during the luting process. The excessive cement was LED light-cured (Elipar S10, 3 M Deutschland GmbH, Neuss, Germany) in all directions for 3 to 5 s at 1200 mW/cm^2^ and then the hardened cement was removed with a scaling instrument. In order to prevent an oxygen inhibition layer a glycerine gel (Liquid Strip, Ivoclar Vivadent AG, Schaan, Liechtenstein) was applied on the adhesive gap before light polymerizing for 30 more seconds. After 24 h of self-curing at room temperature (23 °C), coarse cement residues were removed with a steam cleaner (Triton SLA, Bremer Goldschlägerei Wilh. Herbst GmbH & Co. KG, Bremen, Germany) and the adhesive gap was polished with ceramic polishers at max. 5000 rpm.

### Thermocycling and pull-off tensile load measurement

**Fig. 3:**
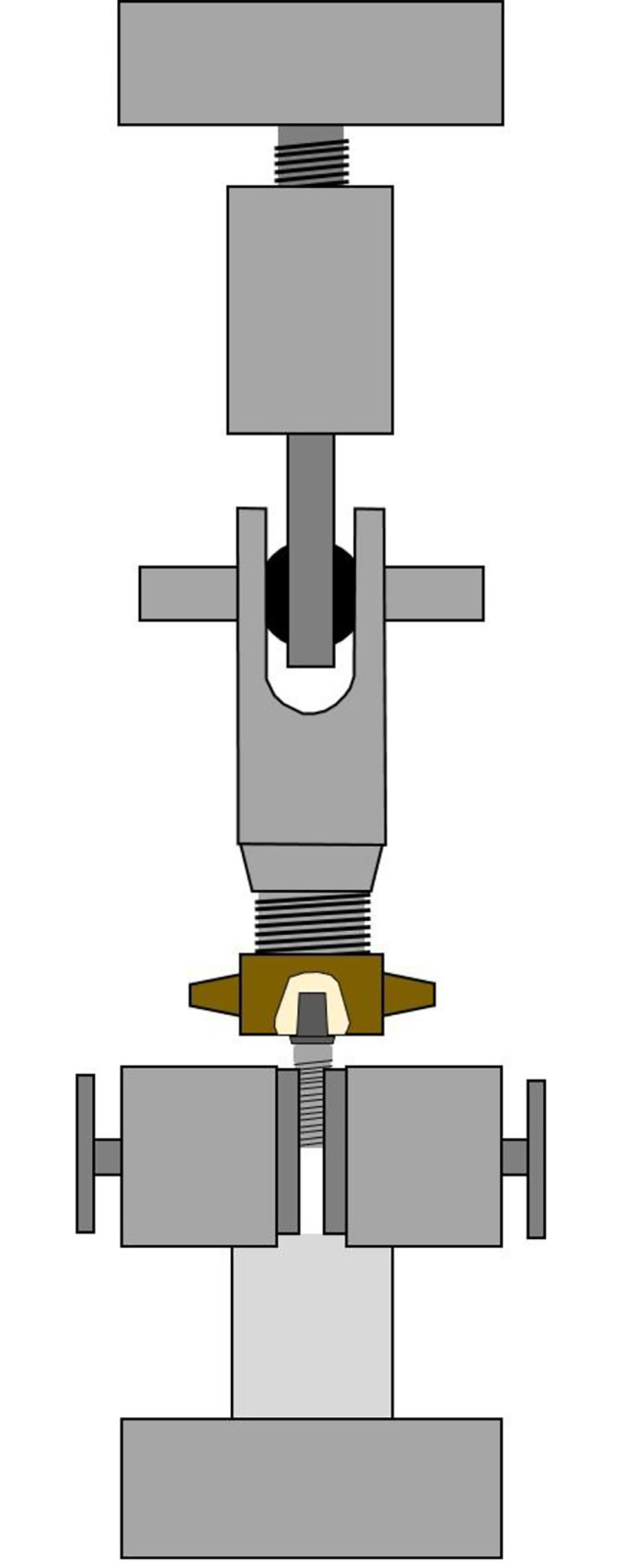
Technical drawing of the pull-off unit. At the bottom the wedge grip specimen holder with the implant and the Ti-Base-Crown-Complex. At the top, the load cell which is connected to the crown by a custom-made jig including a ball-joint

All samples were thermocycled for artificial aging (Thermocycler Willytec, SD Mechatronik GmbH, Feldkirchen-Westerham, Germany). This process was carried out automatically for 5000 cycles at 5 and 55 °C. After 30 s of dwelling per bath, the intervals between the water baths comprised 5 s of dripping off and 5 s of transfer. Until further processing the specimens were stored in distilled water at 23 °C. Pull-off tensile load (TL) measurement was performed in a universal testing machine (Zwick 1425, ZwickRoell GmbH & Co. KG, Ulm, Germany) with a vertical pull-off test. Therefore, an implant (NobelParallel Conical Connection RP 4.3 × 11.5 mm, Nobel Biocare Services AG, Zürich, Switzerland) was fixed in the lower part of the pull-off unit using a wedge grip specimen holder. The hybrid abutment crowns were then successively screwed onto the implant with a tightening torque of 35 Ncm. Afterwards, the crowns were connected to the movable upper part of the pull-off unit using a custom-made jig (Fig. 3) which connected the load cell with the crown by a ball joint so that the force for the pull-off test could be applied in line with the axis. The TL was measured at a crosshead speed of 1.0 mm/min until the crown completely debonded from the titanium base.

### Failure mode analysis

After the TL test, the surfaces of both components were examined with an optical microscope (VHX-1000, Keyence Deutschland GmbH, Neu-Isenburg, Germany) at a magnification of 30x. Failures were categorized as adhesive failures between the surfaces and the resin cement, cohesive failures within the resin cement and both adhesive and cohesive failures simultaneously. Adhesive failures were categorized in complete adhesion of resin cement to titanium, complete adhesion to zirconia or mixed adhesion to both titanium and zirconia.

### Statistical analysis

Statistical analysis was performed with software program SPSS (SPSS Statistics V27, IBM Corp., Chicago, IL, USA) and SAS (SAS 9.4., SAS Visual Statistics, 100 SAS Campus Drive, Cary, NC, USA). In the beginning, descriptive analysis followed by Shapiro–Wilk and Levene tests was performed to check for violation of the assumptions of normal distribution and variance homogeneity. To evaluate the impact of different surface treatments and their combinations on pull-off tensile load, especially (additional) CAP treatment, three-way ANOVA and post-hoc Tukey tests (p = 0.05) were used. The correlation between different surface treatments and failure modes was investigated with Fisher’s exact test (p = 0.05).

## Results

### Pull-off tensile load values

**Fig. 4:**
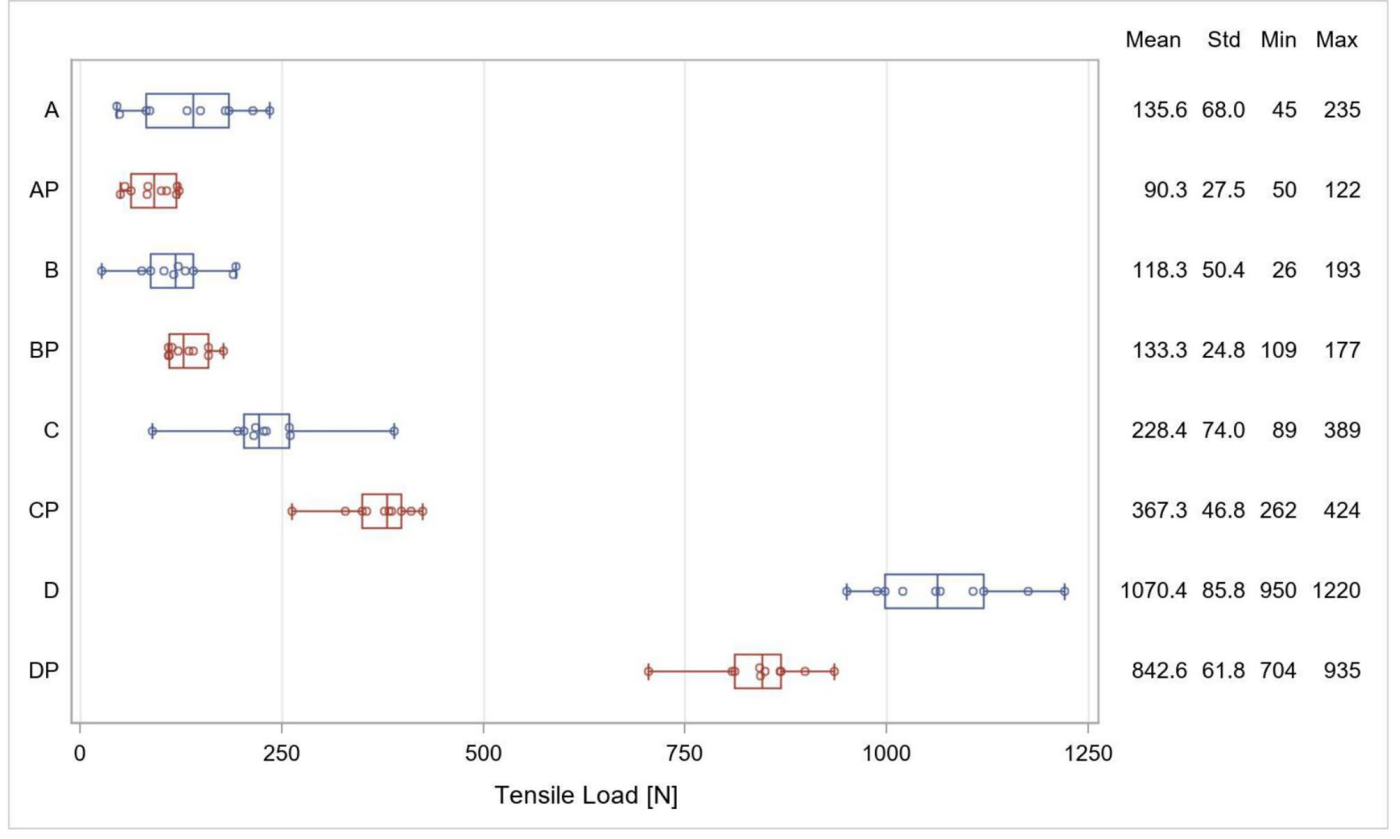
Distribution of the TL values in each group, mean values, standard deviation (Std), minimum and maximum values

The distribution of the TL values of each group is displayed in Fig. 4 and the p-values and differences in the TL for all pairwise comparisons are shown in Table 2. Main effects for primer (p < 0.0001), sandblasting (p < 0.0001) and plasma treatment (p = 0.0257) were significant. Significant interactions were observed between sandblasting and primer (p < 0.0001) as well as between sandblasting and plasma treatment (p < 0.0001). Three-factor interactions were also significant (p < 0.0001).

Group D had significantly higher TL values than all other groups (p < 0.0001). Comparing the treatment procedures with and without additional plasma treatment respectively, the TL values in groups A and AP as well as in groups B and BP showed no significant difference (p > 0.05). In group DP plasma treatment had a negative effect on the TL in comparison to group D (p < 0.0001), whereas in group CP it caused a significant increase in the TL compared to group C (p < 0.0001) which still was significantly lower than the TL in groups D and DP (p < 0.0001).

**Table 2:** P-values and mean differences for pairwise comparisons between groups. P-values are given in the lower triangle of the matrix. The upper triangle contains the differences computed as column mean minus row mean [N]

p	A	AP	B	BP	C	CP	D	DP
**A**	-	-45.3	-17.3	-2.3	92.8	231.7	934.8	707.0
**AP**	0.6669	-	28.0	43.0	138.1	277.0	980.1	752.3
**B**	0.9977	0.9609	-	15.0	110.1	249.0	952.1	724.3
**BP**	1.0000	0.7224	0.9991	-	95.1	234.0	937.1	709.3
**C**	0.0151	< 0.0001	0.0018	0.0115	-	138.9	842.0	614.2
**CP**	< 0.0001	< 0.0001	< 0.0001	< 0.0001	< 0.0001	-	703.1	475.3
**D**	< 0.0001	< 0.0001	< 0.0001	< 0.0001	< 0.0001	< 0.0001	-	-227.0
**DP**	< 0.0001	< 0.0001	< 0.0001	< 0.0001	< 0.0001	< 0.0001	< 0.0001	-

### Failure mode analysis

**Fig. 5:**
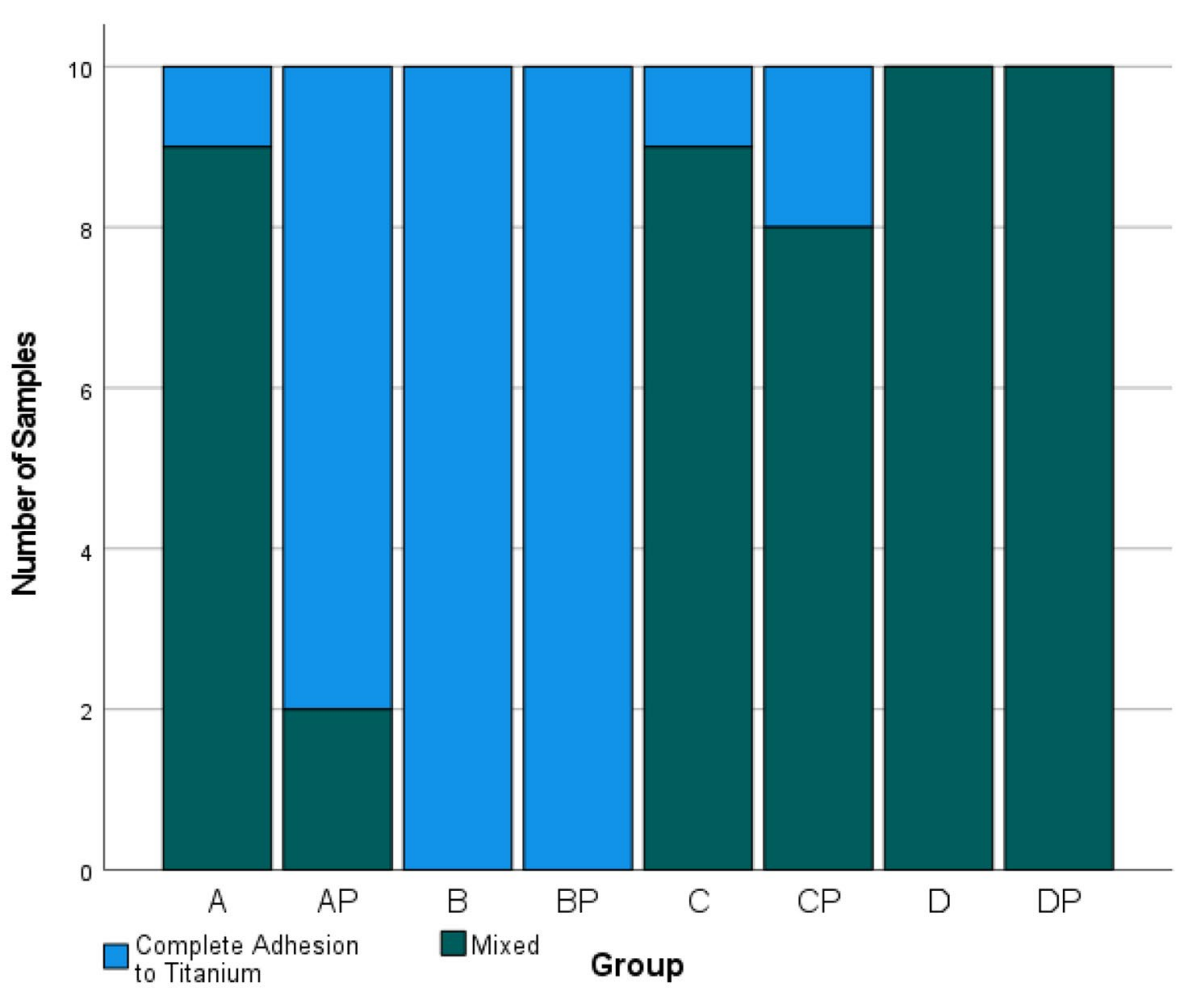
Distribution of failure modes in number of appearances after pull-off test

**Fig. 6:**
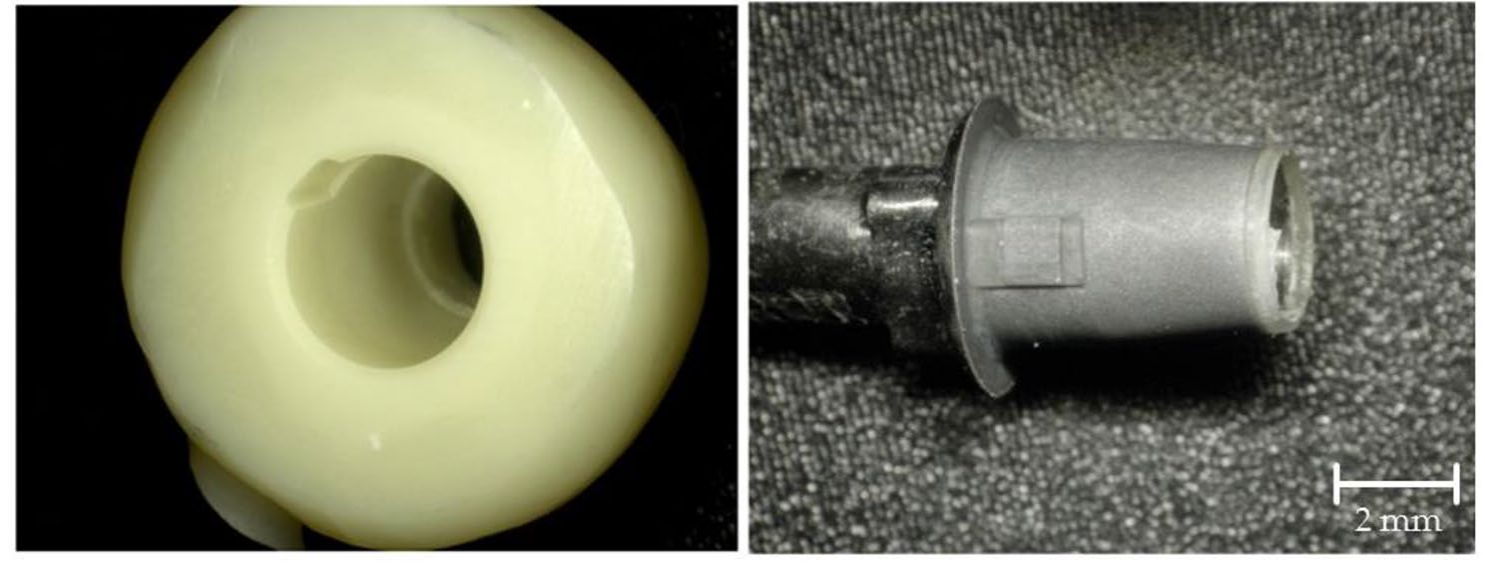
Representative sample for complete adhesion of resin cement to titanium base

**Fig. 7:**
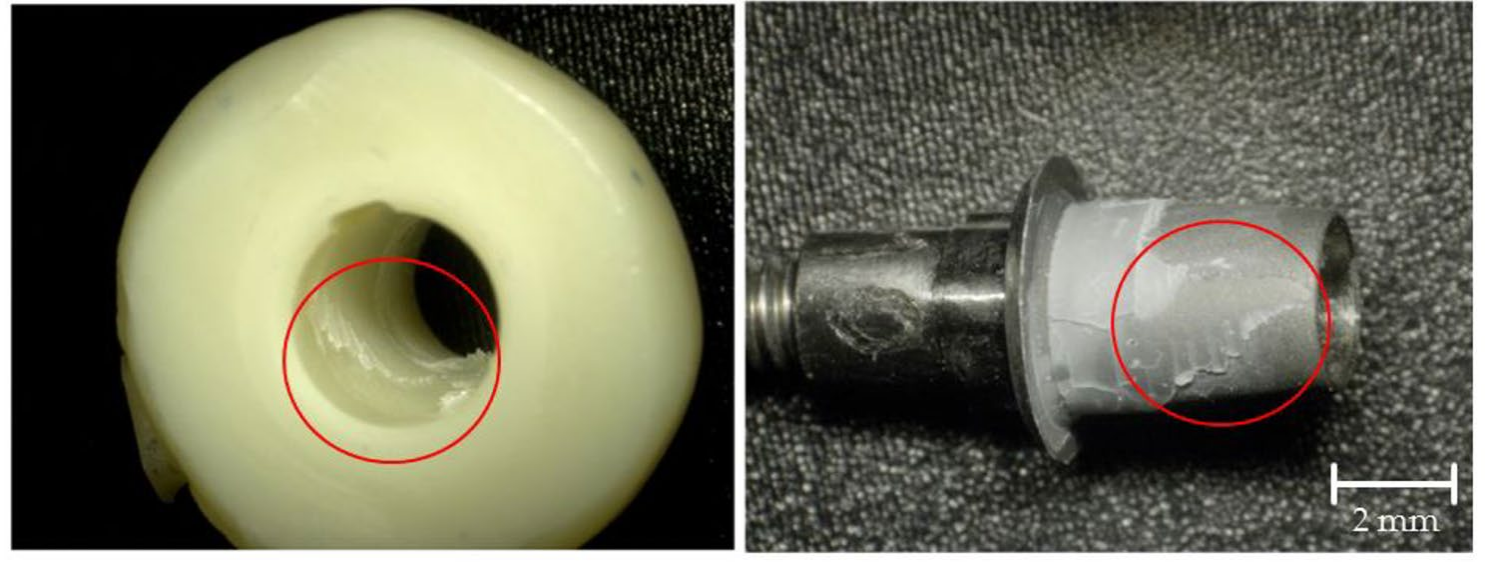
Representative sample for mixed adhesive fractures. The red circles show the resin cement partially remaining on the titanium surface (right) with the corresponding area free of resin cement on the crown (left)

Figure 5 shows the distribution of failure modes. Cohesive failure of the resin cement, as well as adhesive failure between the titanium base and the resin cement with complete adhesion to the zirconia crown, did not occur. A significant correlation was found between the surface treatment protocol and failure mode (p < 0.001). In groups B and BP the resin cement remained completely at the titanium base. Groups D and DP showed mixed adhesive failure in all samples in which the cement at least partially remained at the zirconia crown. While, in groups A and C, the resin cement remained completely at the titanium surface in only one sample each, group CP showed only one more sample with complete adhesion to the titanium surface whereas in group AP this was the case in eight of ten samples. Figures 6 and 7 represent samples of both occurring failure modes under a magnification of 30x.

## Discussion

The present study showed that the pull-off tensile load (TL) in two-piece abutment crowns significantly depends on the surface treatment protocol of the abutment components (p < 0.0001). Looking at the groups with single treatment first, mere sandblasting and mere plasma treatment had no significant effect on the TL in comparison to no treatment (group B vs. A and group AP vs. A). It is noticeable that in all samples of group B and 80% of the samples in group AP adhesion completely failed at the zirconia crown, while without any pretreatment (group A) in 90% of the cases, mixed adhesive fractures were observed. Therefore, mere plasma application as well as sandblasting without further treatment seems most likely to have a negative effect on the adhesion between the resin cement and the zirconia interface which could explain the low tensile load values. In the present study, sandblasting was carried out with 50 μm of Al_2_O_3_ under a pressure of 1.0 bar which conformed to the manufacturer’s specifications for both titanium bases and zirconia crowns. However, for the mere sandblasting of titanium surfaces, Fonseca et al. recommend the largest possible particle size of Al_2_O_3_ up to 250 μm under a pressure of 4.8 bar [19], because smaller sizes could not guarantee sufficient pull-off tensile load which could explain the comparatively low TL values in group B. For zirconia surfaces, only small particle sizes and low pressures up to a maximum of 2.0 bar are recommended due to the risk of local phase transformations and loss of stability [20–23]. Furthermore, sandblasting of zirconia is best combined with chemical treatment such as 10-MDP primer application [11].

Mere primer application increased the TL significantly in comparison to no treatment (p = 0.0151), although the failure modes did not change. This can best be explained by the fact that in group A, the adhesion to the untreated titanium surface was weaker than the adhesion to the untreated zirconia crown. However, at this point it cannot clearly be stated whether the bond of resin cement to titanium was also improved due to the usage of primer. Furthermore, group C showed a significantly higher TL than group B (p = 0.0018) and mainly mixed adhesive fractures, possibly because primer application influences the bond between resin cement and zirconia more positively than sandblasting and therefore shows higher TL values.

However, in some cases the combination of different treatments affected the TL by being higher than just the sum of the individual effects of each treatment. These effects were modified by interactions.

The strongest effect was seen within the combination of sandblasting and primer (group D, conventional protocol), resulting in a significant increase compared to sandblasting alone (group D vs. B) and primer alone (group D vs. C). Furthermore, the combination of all three treatments showed high effects which resulted in increased TL values compared to sandblasting and plasma combined (group DP vs. BP) and to primer and plasma combined (group DP vs. CP). Plasma treatment in combination with primer application had a weaker effect on the TL but still significantly increased it in comparison to mere primer (group CP vs. C) or mere plasma application (group CP vs. AP). When combining plasma with sandblasting and primer, the TL values even decreased significantly in comparison to sandblasting and primer (group D vs. DP). The combination of sandblasting and primer (conventional protocol) showed the largest interaction-effect and the highest tensile load. The conventional protocol has also been approved by many other studies for zirconia [24, 25] as well as for titanium surfaces compared to no treatment or only sandblasting [19, 26]. Fonseca et al. showed that additional adhesive primer application on 50-µm sandblasted titanium surfaces causes a significant increase in shear bond strength (SBS) and is as effective as mere sandblasting with 250 μm of Al_2_O_3_ [19]. Von Maltzahn et al. could find the highest retentive forces in samples where both abutment components were treated in the same way using the conventional protocol as well (sandblasting with 110 μm, 2.0 bar and 10-MDP primer) [8], even though the TL values were lower than those in the present study. This could possibly be explained by the different particle sizes of Al_2_O_3_ and higher pressure (110 μm vs. 50 μm; 2.0 bar vs. 1.0 bar). Studies have shown that, when combining sandblasting with primer application, a particle size of 50 μm [12, 27] and a pressure of 1.0 bar [23] produce the best TL values.

However, not only in group D primer application led to higher TL values. In direct comparison, all groups with additional primer treatment showed significantly increased TL values (p < 0.001). It is known that primers, especially primers containing the amphiphilic phosphate monomer 10-MDP, have a huge impact on the adhesion of resin cements to zirconia [28–31]. In groups AP, B and BP (without primer), adhesion fractures mainly appeared on the zirconia crown which means that the adhesion of the resin cement to zirconia was weaker than to titanium. Only in group A, despite low TL values, there were mostly mixed adhesive fractures which did not appear to be due to acceptable adhesion between the resin cement and zirconia, but rather to even weaker adhesion to the unconditioned titanium surface. However, in groups C, CP, D and DP (with primer) mixed adhesive fractures, in which the cement at least partially remained at the zirconia crown, could be observed in almost every sample. Therefore, it can be confirmed that adhesion to zirconia seems to be increased by primer application.

A significant correlation was found between the surface treatment protocol and failure mode (p < 0.001), as well as significant differences between mean TL values and the localization of cement residues, regardless of the surface treatment (Mann–Whitney U test: p < 0.001). In samples with complete adhesion to the titanium surface (n = 32), mean TL values were 132.4 N (95% CI [103.4 to 161.4 N]), while, in samples with a mixed fracture pattern (n = 48), they were 533.9 N (95% CI [423.3 to 644.4 N]). Nevertheless, there was no sample with complete adhesion of the resin cement to zirconia. It remains questionable whether this would mean a further increase in TL values. The conclusion is that TL is determined by the bond between the resin cement and the zirconia surface which in turn depends on the surface treatment of the ceramic interface.

The pretreatment of zirconia is compromised due to different material properties. In order to protect the ceramic surface and reduce unforeseen complications, or to avoid the use of potentially toxic chemicals, the search for alternative methods seems to be justified. Nevertheless, the high TL values of the conventional protocol place some demands on the new method which should at least be equivalent. The present study therefore investigated the impact of CAP on TL in two-piece abutment crowns in combination with other treatments considering whether CAP could supplement or even replace one of the common methods or all of them.

The failure modes of groups BP and DP, in comparison to groups B and D, show that plasma treatment seems to have no supporting effect on the adhesion between resin cement and zirconia. Only group CP showed significantly higher TL than group C (p < 0.0001), without a significant change in failure mode. This could be explained by the assumption that CAP increases the surface wettability of titanium as well as of zirconia and therefore causes the primer to be more sufficiently distributed on the surface. However, the TL in group CP was still significantly lower than the TL in group D (p < 0.0001). Comparing all CAP groups with group D, it can be stated that CAP cannot support or even replace any common pretreatment method.

Furthermore, two successive but related studies by Ahn and Kim et al. showed that the SBS between Bis-GMA/TEGDMA resin cement and zirconia after thermocycling was highest when conditioned under the conventional protocol (sandblasting and primer application, comparable to the treatment of group D in the present study) [17, 32]. Additional CAP treatment (comparable to the treatment of groups AP, BP, CP and DP in the present study) had a significantly negative impact on SBS in all groups. In the present study, only in group DP in comparison to group D CAP had a significantly negative influence on TL. In all other groups, it had no significant impact or even could significantly increase TL. The CAP used in these two studies was operated with argon gas for 60 s which is much more aggressive to the surface than CAP operated with ambient air for 15 s, and it has only been applied to the zirconia surface. Therefore, it remains questionable whether the results of the present study are comparable to those above mentioned.

Recently, Kim et al. examined the TL between sandblasted titanium bases and differently conditioned zirconia crowns, focusing on the combination of CAP and 10-MDP primer application [18]. They also found no significant difference between the non-treatment group and the CAP-only group (similar to groups A and AP in the present study). Furthermore, comparing the primer-only and the primer-and-CAP group (similar to groups C and CP in the present study), CAP even led to significantly lower TL values which differs from the results of the present study. However, in their study as well only the zirconia surface was treated with different combinations of CAP. The titanium bases in all groups were only sandblasted. The influence of CAP on the titanium surface in this two-piece abutment complex was therefore not examined here, and this could explain the differences in results. Assuming that the adhesion between the resin cement and the titanium surface, and therefore the TL values, is improved by CAP prior to primer application could explain the different results.

## Conclusion

Provided that the titanium and zirconia surfaces in two-piece abutment crowns are pretreated in the same way, the following conclusions can be drawn. Within the single treatment groups, only the application of primer has a significant effect on the TL. Sandblasting and CAP alone seem to have a negative impact on the adhesion of resin cement to zirconia. Furthermore, within the groups that combine two or three treatments, the combination of primer and sandblasting shows the largest effect on tensile load.

Within the limitations of this study, it can be stated that sandblasting followed by chemical treatment with primer is the most appropriate surface treatment protocol for both zirconia and titanium surfaces in two-piece abutment crowns. In this study, CAP was not able to support or even replace any common pretreatment method. Further investigations regarding other plasma systems, such as low-pressure plasma or inert gas plasma, and their combination with different adhesive systems are necessary.

## Data Availability

The datasets used and/or analyzed during the current study are available from the corresponding author on reasonable request.

## Abbreviations

10-MDP: 10-Methacryloyloxydecyldihydrogen-phosphate
Bis-GMA: Bisphenol A-Glycidyl Methacrylate
CAD: Computer-Aided-Design
CAM: Computer-Aided-Manufacturing
CAP: Cold Atmospheric-Pressure Plasma
CEREC: CERamic REConstruction
SB: Sandblasting
SBS: Shear Bond Strength
Std: Standard Deviation
TEGDMA: Triethylenglycoldimethacrylate
TL: Pull-Off Tensile Load
